# Supplementary material for: Circulating Tumor Cells and TWIST Expression in Patients with Metastatic Gastric Cancer: A Preliminary Study
Source: J Clin Med. 2021 Sep 29;10(19):4481. doi: 10.3390/jcm10194481 (PMC8509658; doi:10.3390/jcm10194481)
Supplement: Supplementary file 1 [file jcm-10-04481-s001.zip › jcm-1397054-supplementary.pdf]

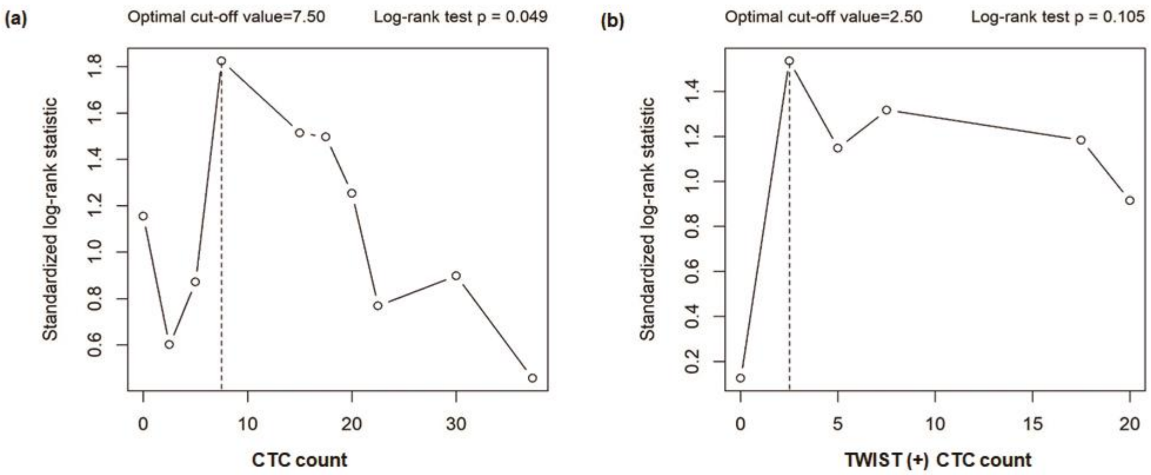

Supplement Figure S1. Determination of optical cut-off points using the maximally selected rank statistic. (**a**) Circulating tumor cells. (**b**) TWIST (+) circulating tumor cells.
